# Advanced biopolymer nanocomposites for real-time biosurveillance and defense against antimicrobial resistance and viral threats

**DOI:** 10.1039/d5ra04504e

**Published:** 2025-09-10

**Authors:** Ashwag S. Alzahrani, Khalid A. Alamry, Mahmoud A. Hussein

**Affiliations:** a Chemistry Department, Faculty of Science, King Abdulaziz University Jeddah 21589 Saudi Arabia kaalamri@kau.edu.sa maabdo@kau.edu.sa mahussein74@yahoo.com; b Chemistry Department, Faculty of Science, Assiut University Assiut 71516 Egypt

## Abstract

Antimicrobial resistance (AMR) presents an ever increasing challenge to health globally. Until now, all conventional antibiotics and antivirals are struggling to keep up with minimum efficacy and eradication rate in both civilian and military contexts. The purpose of this review is to compile all current research on the use of functionalized biopolymer nanocomposites, highlighting them as the next-generation of antimicrobial platforms. The emphasis is placed on natural and synthetic biopolymers such as chitosan, alginate, cellulose, and polyvinyl alcohol, all of which are engineered with a range of nanofillers. The nanofillers themselves include silver, zinc oxide, copper oxide, titanium dioxide, graphene derivatives, and metal–organic frameworks. The synergy of these materials not only enhances but broadens antimicrobial activity. Key mechanisms underlying AMR have been elucidated. These include but are not limited to genetic mutation, efflux pump activation, enzymatic inactivation, and viral adaptation. Besides this the multifaceted antimicrobial actions imparted by biopolymer–nanofiller composites have also been thoroughly described. Additionally, chemical modifications such as sulfation, carboxymethylation, and amination are discussed as critical strategies to further improve antimicrobial efficacy. Regarding practicality, real-world applications including wound care, medical device coatings, air filtration, packaging, biosensors, and military-grade protective equipment have been presented along with all pros and cons as well as limitations and challenges. As for drawbacks, such as toxicity, scalability, regulatory considerations, and the potential for environmental impact, this paper has attempted to critically evaluate these and provide directions for future research.

## Abstract 2

The work is focussed on the use of all advanced functionalized biopolymer nanocomposites for real-time biosurveillance and defense against antimicrobial resistance and viral threats, which present an ever-increasing challenge to health globally.

## Graphical abstract

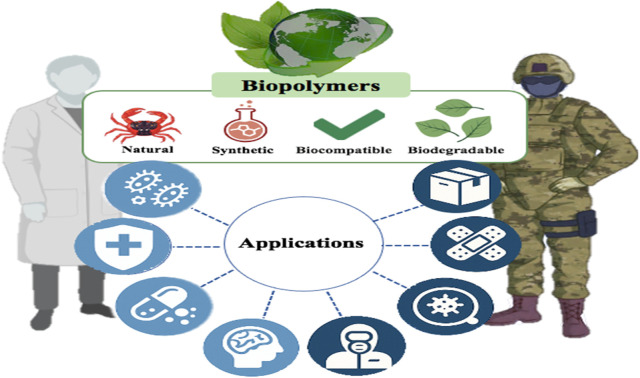

## 1. Introduction

In recent years, emerging and re-emerging infectious diseases have posed mounting challenges to global public health systems.^1^ Among these challenges are critical issues for national security and modern healthcare infrastructure, foremost among them being resistance to antibiotic treatment in bacterial and viral pathogens.^2^ Resistant microbial strains have emerged primarily because of the improper use of antibiotics and antiviral drugs, particularly in veterinary, therapeutic, and agricultural settings.^3^ Bacterial pathogens such as methicillin-resistant *Staphylococcus aureus* (MRSA), Carbapenem-Resistant Enterobacteriaceae (CRE), multidrug-resistant *Pseudomonas*, rapidly evolving strains of SARS-CoV-2; and several other viruses threaten present-day therapies and expose flaws in global readiness to combat the ever-evolving landscape of microbes.^4^ This is even more pertinent in extremely vulnerable settings, such as congested communities, war zones, and military facilities. Emerging viral hazards, antibiotic resistance (AMR), and bioterrorism are occasionally considered possible threats to national and global security.^5,6^ With little to no access to rapid and efficient medical care, defense operations involving soldiers bear the brunt of biological hazards.^7^ Soldiers or security units are exposed to polluted environments and experience wounds that are open to infection.

To address these escalating threats, researchers have developed new antimicrobial materials that are not based on conventional drug-based mechanisms.^8^ The tactical advantages of these hybrid materials, including chemical versatility, biocompatibility, biodegradability, potential for real-time biosensing applications, and increased effectiveness against rising resistance, overshadow their drawbacks. Extensive studies have been conducted on the natural antibacterial and antiviral capabilities of biopolymers, including chitosan, sulfated polysaccharides (such as ulvan, fucoidan, and carrageenan), alginate, cellulose derivatives (such as carboxymethyl cellulose), polylactic acid, and polycaprolactone.^9–11^ The inherent activity of biopolymers allows researchers to chemically alter specific functional groups through targeted chemical modifications. Sulfation, carboxymethylation, phosphorylation, enzymatic grafting, and crosslinking with biocompatible compounds are methods that significantly enhance antimicrobial activity, mechanical stability, and degradation resistance.^12–14^ Additionally, biopolymers are sometimes supplemented with nanofillers, which further enhance their antibacterial potency.^15^ These comprise metal-based nanoparticles, such as silver nanoparticles, zinc oxide, copper oxide, and titanium dioxide. Other unique elemental combinations include carbon-based nanomaterials such as graphene, carbon dots, and fullerenes.^16^ Polymeric nanostructures and metal–organic frameworks also contribute to this process.^17,18^

In the military and defense spheres, there is a pressing need for tools and equipment that can counter growing microbial resistance, especially in areas of armed conflict.^19^ These include personal protective equipment, portable water-filtering systems, protective medical fabrics, self-sterilizing devices, and antimicrobial field-ready dressings.^20^ Government and military agencies have increasingly invested in growing global concerns regarding AMR and viral threats to conduct additional research and fund more studies on protective equipment.^7^ Agencies, including the Centers for Disease Control and Prevention (CDC), the World Health Organization (WHO), and other defense research teams, emphasize the need for a counterstrategy against biothreats and epidemics.^21–23^ And despite the promise of biopolymer applications, a multitude of hurdles remain to be addressed. These include biosafety, standardization, scale-up and cost-effectiveness to name a few. In addition, for effective deployment, it is essential that material formulation be optimized, and regulatory frameworks be developed. This is also why interdisciplinary collaboration among materials scientists, microbiologists, engineers, and military experts is critical for accelerating the transfer of these technologies from lab-scale innovations to field-ready solutions.

This study provides a detailed review of the role of biopolymer-based nanocomposites in combating viral challenges and antibiotic resistance. The main emphasis is on chemical modifications, nanofillers, and different biopolymers, as well as their practical applications in military defense, real-time biosurveillance, and infection control. This study aims to link materials science, microbiology, and military policy by integrating knowledge from recent technologies and studies.

## 2. Mechanisms of antimicrobial and viral resistance

### 2.1 Bacterial resistance mechanisms: mutation, efflux, biofilms, and genetic exchange

Inefficiencies in antimicrobial therapy, such as inappropriate dosing, poor compliance, overuse, or weak formulations, have enabled bacterial pathogens to circumvent antibiotic effects through multiple complex strategies.^24^ This has gradually led to the emergence and active prevalence of resistant strains, which has seen a spike in recent years.^25^ This sudden increase reflects the failure of both healthcare providers and patients to address the situation appropriately.^26^ Bacteria employ a multitude of mechanisms to resist modern antimicrobial drugs, including spontaneous genetic mutations, alteration of drug targets, active drug efflux, biofilm formation, horizontal transfer of resistance genes across microbial communities, and enzymatic degradation of drugs.^27^ A summary of the results is provided in Table 1.

**Table 1:** Summary of mechanisms of antimicrobial and viral resistance

Mechanism	Pathogen type	Details	Example	Ref.
Random mutation	Bacteria	Spontaneous or induced changes in bacterial genome to reduce drug-target binding	Fluoroquinolone resistance *via* gyrA mutations	31
Efflux pump activation	Bacteria	Active expulsion of antibiotics from the cell to lower intracellular concentrations	AcrAB-TolC system in *E. coli*; NorA in *S. aureus*	36
Biofilm formation	Bacteria	Close population that resists antibiotics *via* EPS matrix and altered metabolism	*Pseudomonas aeruginosa* biofilms in wounds	38
Enzymatic inactivation	Bacteria	Production of enzymes that break down antibiotics	NDM-1 and OXA-48 carbapenemases in *Klebsiella*	51
Alteration of targets	Bacteria	Structural modification of binding sites to prevent drug interaction	mecA gene in MRSA; methylation in macrolide resistance	32
Horizontal gene transfer	Bacteria	Transfer of resistance genes *via* plasmids, integrons, or transposons	Plasmid-mediated ESBL production in *E. coli*	41
Hypermutation	Virus	RNA viruses accumulate mutations rapidly due to lack of proofreading polymerases	HIV reverse transcriptase mutation M184V	80
Recombination/reassortment	Virus	Exchange of genome segments between viruses	Influenza A reassortment; coronavirus recombination	59
Quasispecies & selective pressure	Virus	Variant swarms which can adapt under selective pressure	HCV quasispecies variation; HIV treatment escape	79
Immune evasion	Virus	Multiple mechanisms involved in this	Omicron variant mutations (*e.g.*, K417N, P681H)	86

#### 2.1.1 Mutation-based resistance

Random mutations in bacterial genomes are common, especially in genes encoding antibiotic targets.^28^ When these are altered, it substantially reduces the binding affinity of drugs, thereby improving the chances of survival for the pathogen.^29^ Sublethal doses have been shown to cause point mutations in bacteria. This work was performed using the SOS system, which is a stress response to DNA damage. This leads to the induction of error-prone DNA polymerases, which increases the overall rate of mutation; in other words, placing the bacteria in a state of hypermutation.^30^ Some examples of mutation-based resistance include gyrA and parC genes,^31^ which, if affected, lead to fluoroquinolone resistance, or ribosomal mutations such as methylation that lead to macrolide and aminoglycoside resistance.^32,33^ Sometimes, there are direct modifications in the protein structures of bacteria, such as penicillin-binding proteins (PBPs); if these are altered, they confer β-lactam resistance.^34^

#### 2.1.2 Efflux pump activation

A common mechanism involves the efflux pumps. These transmembrane proteins constantly flush out any antibiotics that penetrate the bacteria.^35^ Thus, bacterial cells reduce the intracellular drug concentration to subtherapeutic levels. The pump does not necessarily have to be selective, as there are occurrences of ‘broad-spectrum’ pumps in their targets, such as AcrAB-TolC in Gram-negative bacteria or NorA in *Staphylococcus aureus*, which can expel structurally diverse antibiotics, effectively creating resistance to multiple classes of drugs.^36,37^

#### 2.1.3 Biofilm formation

One strategy that requires the collective effort of multiple bacteria is biofilm formation.^38^ These are highly organized bacterial communities that shield themselves by producing a gel-like covering.^39^ The extracellular polymeric substance (EPS) produced by bacteria protects the community at the cost of placing pathogens in a quasi-dormant metabolic state, where inner functions are suppressed.^40^ The EPS matrix not only makes antibiotic penetration more difficult but also allows cells to focus on delicate activities such as horizontal gene transfer (HGT).^41^ This unchecked transfer is a major contributor to the ever-increasing rates of resistance.^42^ Even distantly related bacterial species can take advantage of HGT, thereby enabling the rapid spread of multidrug-resistant organisms across humans, animals, and environmental reservoirs.^43,44^ Noteworthy examples include *Pseudomonas aeruginosa*, *Staphylococcus aureus*, and *Escherichia coli*, which have been identified in biofilms formed within infected wounds, catheters, and ventilator-bound patients.^45–47^

#### 2.1.4 Enzymatic inactivation of antibiotics

Another critical bacterial strategy is the production of enzymes that deform antibiotics, whether by chemical degradation or subtle modification, before the drugs reach their destination.^48^ The best-known examples, often highlighted in textbooks, are β-lactamases. Since the use of penicillins became widespread, this was one of the first forms of resistance discussed in the literature.^49^ These enzymes hydrolyze the β-lactam ring of penicillins and cephalosporins, rendering them inactive.^50^ Earlier β-lactamases were narrower in their target of action, but this was soon followed by extended-spectrum β-lactamases (ESBLs) and carbapenemases, both of which can hydrolyze a broader array of β-lactams, including third-generation cephalosporins and carbapenems. This upgrade has made these types of bacteria more notorious in hospital settings, limiting options for the management of patients prone to nosocomial infections.^51,52^

Other drugs, such as aminoglycosides, have become less dependable, but laboratory efforts are still ongoing to find ways to counter resistance to this class of drugs. One study discussed how small-molecule inhibitors of resistance can generated by enzymatic modification of aminoglycoside drugs.^53^ Whether this same approach can work in for other classes of drugs or whether bacteria can evolve further to nullify these efforts is worth investigating in future studies.

#### 2.1.5 Environmental stress and resistance amplification

**Fig. 1:**
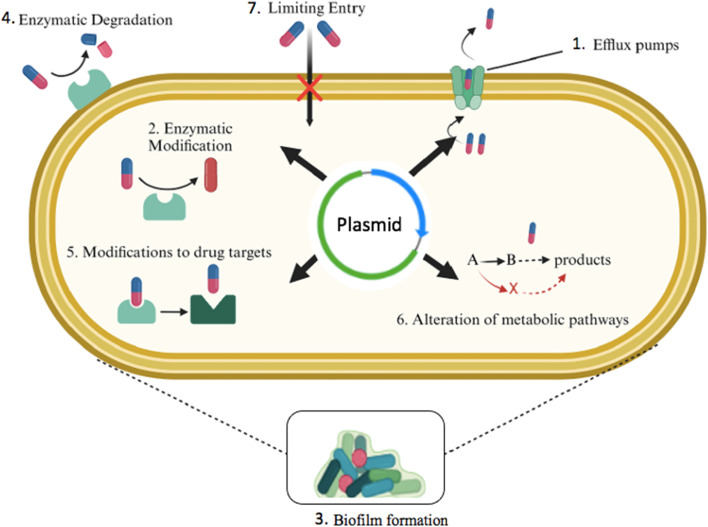
Schematic representation of the main mechanisms of AMR.

The human body is not the only reservoir targeted by antimicrobial therapy. To aid in preventive measures, antibiotics are also used in clinical, agricultural, and wastewater settings. However, if strategies do not align with recommended guidelines, such as the use of subtherapeutic levels, or if there is failure to control drug disposal, leading to excessive contamination of the environment, this only further exacerbates bacterial resistance.^54^ One study by Bagra *et al.* reported that rivers contaminated with Cu ions, which served as a stressor to natural biofilms, allowed for the invasion of antimicrobial-resistant *E. coli*.^55^ Environmental stress provides bacteria with opportunities to survive, mutate, and engage in HGT. Additionally, co-selection is caused by the use of heavy metals and biocides.^56^Fig. 1 summarizes these resistance mechanisms.

### 2.2 Mechanisms of viral evolution and drug resistance

Viral evolution is an ever-changing process influenced by several molecular and ecological forces responsible for the increase in drug resistance and immune escape. Viruses survive due to their versatility, adapting to different selective pressures. There are several mechanisms through which viruses evade host immune responses or antiviral therapies. These include high levels of mutation rates and recombination events; additionally, certain antivirals can put selective pressure and certain species of viruses are known to have error-prone replication.^57^

#### 2.2.1 High mutation rates

One of the foremost contributors to viral resistance is the intrinsically high mutation rates observed in RNA viruses. Certain viruses exhibit a wide range of variability in their biochemical structure. This is particularly true for RNA viruses such as, influenza,^58,59^ HIV-1,^60,61^ hepatitis C virus (HCV),^62^ and SARS-CoV-2.^63^ The significantly high mutation rates can be attributed to the presence of certain enzymes such as RNA-dependent RNA polymerases (RdRps) or reverse transcriptases (RTs), which fail to proofread during the replication process.^64^ This leads to an increased number of point mutations, which in turn leads to the production of abnormal proteins that cannot be accurately targeted by antivirals or the immune system due to a lack of identification or compatibility.^65^ For example, single amino acid substitutions in the neuraminidase enzyme of influenza viruses have allowed them to resist the action of oseltamivir, which is still commonly used to manage the flu. This is only one of the many possible mutations, and the rates of mutation may be higher than those calculated through conventional means,^66^ which raises concerns about whether these pathogens can be controlled in the long term. Long-term viral suppression has not been achieved because of this mutation-driven adaptability, leaving prevalent viruses, such as influenza A and SARS-CoV-2, still a global threat.^67^

#### 2.2.2 Genetic recombination

Novel viral strains with variable phenotypes can come into existence through the process of genetic recombination. To explain the process, two related viruses exchange genetic material during host cell infection. A well-known example is the influenza virus, which causes pandemics. Indeed, the recombination of gene segments within the influenza virus can produce strains with completely reversed antigenicity and drug sensitivity, never seen before.^68^ This mechanism of antigenic shift significantly contributes to immune escape, which can give rise to pandemics, as observed with both influenza and coronaviruses.^69,70^

#### 2.2.3 Selective pressure from antiviral therapy

Subtherapeutic or incomplete antiviral regimens are known to create a habitat where drug-resistant viral strains can grow.^71^ This poses a grave threat in cases involving chronic viral infections, such as HIV, HCV, and COVID, where incomplete suppression of the viruses can lead to resistant strains; this in turn makes management more difficult all the while increasing the morbidity of the patient.^72–74^ Even simple cases of flu can become burdensome for the elderly or immunocompromised when they are refractory to medical management.^75^ One workaround is the use of combination therapies, such as HAART in HIV, which mitigates this risk.^76^

#### 2.2.4 Quasi-species

Quasi-species represent a diverse population of viral variants that coexist within the host. Considering the three aforementioned mechanisms, it is no surprise that all viruses present within a host at any given time do not belong to a single genotype, but rather form a swarm of diverse variants.^77^ This makes the entire swarm or quasi-species robust to changing environmental conditions. However, combination drug therapies can eliminate most of these variants.^78^ Clinically, some symptoms exhibited by different viral genotypes can and often will overlap. Yet, when one considers scenarios such as a patient developing Guillain–Barré syndrome after a common cold *versus* another remaining asymptomatic despite years of HIV-1 infection, the unpredictable behavior of viruses becomes evident.^79^

#### 2.2.5 Resistance at the molecular level

At the molecular level, viruses have devised several precise mechanisms to counteract the actions of antiviral agents. To enlist, these include alteration of the drug-binding sites, switching of the target pathway, and reduction of intracellular drug activation. For example, the HIV virus can undergo mutations such as M184V and K103N, which imparts a property of resistance against nucleoside and non-nucleoside reverse transcriptase inhibitors (NRTIs, NNRTIs), respectively.^80,81^ To illustrate another example, the influenza virus exhibits resistance to M2 ion channel inhibitors such as amantadine^82^ and neuraminidase inhibitors such as oseltamivir by utilizing alternate replication pathways.^83^ Since the finale of the COVID epidemic, the SARS-CoV-2 variants, which arise due to mutations in the spike gene, deserve special mention.^84^ With the rapid emergence of Delta and Omicron, this has underscored the importance and necessity of genomic surveillance.^85^ RT-PCR-based mutation panels and whole genome sequencing have been productive in tracking these evolutionary changes to influence preventive measures and public health responses.^86^

### 2.3 Pathways of transmission in civilian *versus* military environments

Although resistance in bacteria and viruses forms one part of the plight, the other is the mode of transmission, which can be complex. While there is considerable overlap between civilian and military transmission, the latter introduces additional risk factors that can reinforce the spread of the infection.

#### 2.3.1 Civilian transmission pathways

Here, transmission predominantly occurs through direct human-to-human contact, fomites, airborne particles, and exposure to contaminated water or food. Moreover, healthcare-associated infections (HAIs) are a major source of AMR pathogens, including in hospitals or health care facilities that cater to prolonged inpatient care.^87^ At the community level, poor sanitation, urban crowding, and lack of vaccination coverage, as observed in third-world countries, disseminate pathogens at an amplified strength of virulence.^88^ Additionally, certain lifestyle practices that directly affect health negatively (*e.g.*, promiscuous, unprotected, or unnatural sex) coupled with excessive or suboptimal use of antibiotics promote the transmission of resistant organisms very easily.^89^

#### 2.3.2 Military transmission pathways

In comparison, military environments provide ideal nesting grounds for the further amplification of resistant strains. Deployment camps, naval vessels, field hospitals, and forward-operating bases are choked at times with service members working under austere conditions.^90^ This is also the overall decrease in immunity in soldiers who overexert or sustain wounds that contain opportunistic bacterial infections.^91^ Even more concerning is how military personnel returning from deployments or services may act as vectors, introducing novel AMR strains into civilian habitats or healthcare systems.^92^ Viral outbreaks within deployed units have historically been reported, including outbreaks of adenovirus, influenza, norovirus, and, most recently, COVID-19.^93–96^

## 3. Biopolymers for antimicrobial and antiviral applications

**Fig. 2:**
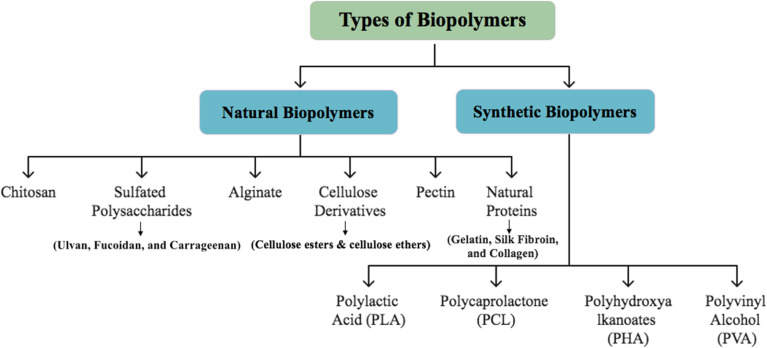
Outlines the classification of biopolymers.

Biopolymers have emerged as attractive materials for antibacterial and antiviral applications owing to their biocompatibility, biodegradability, and functional flexibility. These biopolymers can be broadly divided into two types: natural biopolymers, which are generated from renewable biological sources, and synthetic biopolymers, which are manufactured through controlled polymerization methods to attain certain qualities. This classification is clearly illustrated in (Fig. 2).

Functionalized biopolymers primarily exert antimicrobial action through a number of mechanisms which include disrupting microbial membranes, generating reactive oxygen species, and interfering with biofilm formation.^9–11^ When compared to traditional antibiotics, biopolymers are generally less specific. This broad spectrum of activity is not necessarily a disadvantage as they are less likely to induce resistance. Efficacy-wise, biopolymer-based systems are at least as effective, if not more, than traditional antibiotics *in vitro*. This is especially true against resistant strains. However, for systemic infections, antibiotics remain more established, while biopolymers are mostly applied in topical or device-related settings.

### 3.1 Natural biopolymers

#### 3.1.1 Chitosan

**Fig. 3:**
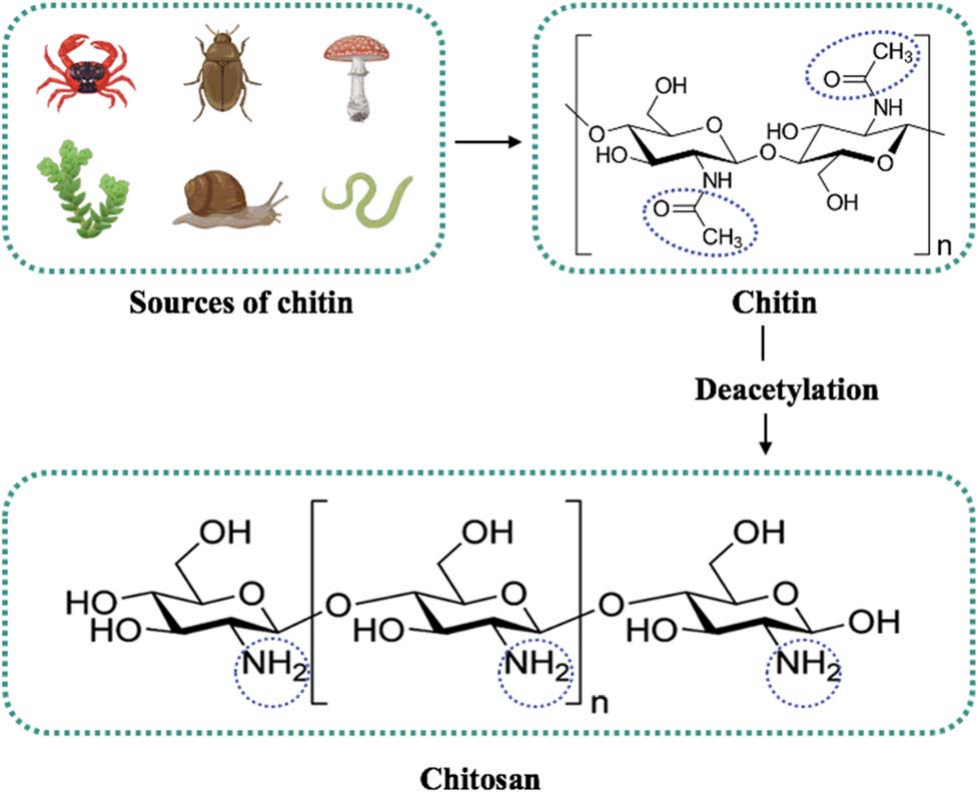
Sources of chitosan and molecular structure of chitin and chitosan. The circles denote the amino groups of chitosan.

Chitosan, derived from chitin, is a group of linear polysaccharides found in the outer covering of certain living organisms, such as exoskeletons of crustaceans, cell walls of fungi, and insect cuticles. Structurally, it is a copolymer with variable acetylation and polymer length and contains amino and hydroxyl functional groups that define both its reactivity and properties (Fig. 3).^9^ Historically, for commercial purposes, it was made by the chemical deacetylation of chitin from crustacean sources and has served a myriad of applications, including agriculture, food industry, pharmacy, cosmetology, medicine, chemistry, textile, and paper industries.^97^

In pharmacy, it has been extensively studied in drug delivery systems; in the food industry, it is used in food processing; in agriculture, it has been used as a bio stimulant and in crop protection.^98^ A particular area of interest surrounding chitosan is that it exhibits broad antimicrobial effects, affecting bacteria, fungi, and viruses through different mechanisms such as chelation, surface interaction, intracellular disruption, and interference with microbial metabolism. The target hosts include Gram-positive bacteria, Gram-negative bacteria, and a few species of fungi such as *Candida*.^99^ Recently, chitosan has been used to tackle AMR. It promises new insights and applications in the subject matter where conventional antibiotics have failed.^100^ The antimicrobial action of chitosan-based biopolymers involves three distinct mechanisms: electrostatic interactions, interactions with bacterial DNA, and chelation of metals.

#### 3.1.2 Sulfated polysaccharide

**Fig. 4:**
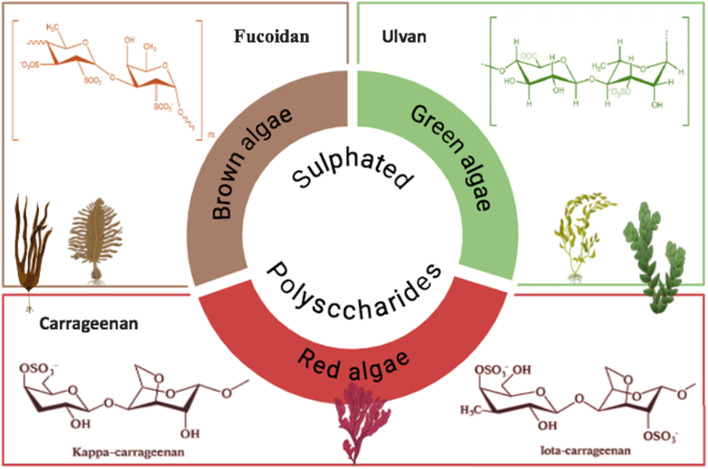
Characteristic backbones of macroalgae sulfated polysaccharides.

Polysaccharides are large chains of simple sugars covalently linked by glycosidic bonds.^101^ Marine macroalgae and seaweed are abundant sources of polysaccharides, with sulfate groups linked to them. Other examples include fucoidan, which is found in brown seaweeds, ulvan which is found in green seaweeds, and carrageenan which is found in red seaweeds (Fig. 4). They find applications in the food, cosmetic, and pharmaceutical industries owing to a number of favorable properties, such as biostability, hydrocolloidal properties, abundant sources, ease of extraction, and purification without the drawback of polluting the environment; they are essentially imperishable and innocuous.^102^

In the food industry, because of their excellent biological properties they are used as food fortifiers, food packaging, supplements, animal feeds, stabilizers, emulsifiers, additives, and thickening agents.^103^ When it comes to AMR toward existing therapies, some bacteria and fungi form a biofilm.^104^ Recently, sulfated polysaccharides have been found to be useful in the treatment of mutant strains of HIV that are resistant to reverse transcriptase and are also employed in medicine delivery systems.^105,106^

#### 3.1.3 Alginate

**Fig. 5:**
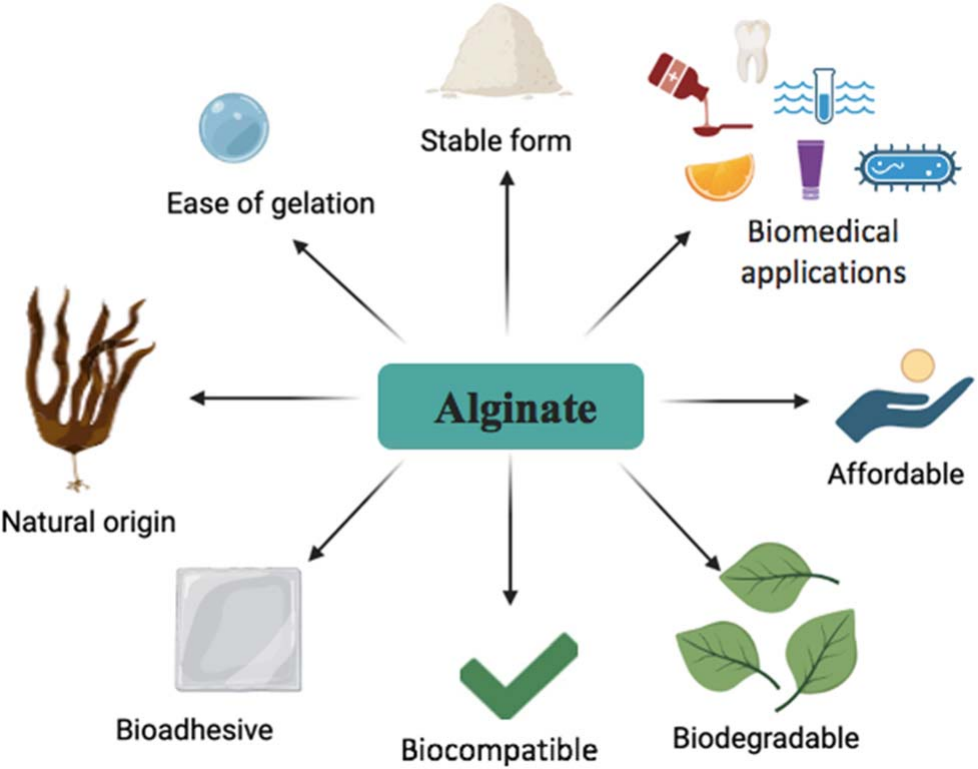
Diagram illustrating the key properties of alginate.

Alginates are salts of alginic acid and are naturally occurring polysaccharides derived from brown seaweeds such as *Macrocystis*, *Ascophyllum*, and *Alario*.^10^ Similar to chitin derivatives and sulfated polysaccharides, alginate has tremendous biomedical applications because of its biocompatibility, non-toxic nature, and affordability (Fig. 5). Alginates also have pharmaceutical applications in addition to being edible enough to be used in food preparations.^107^

In the pharmaceutical industry, alginate dressings are commonly used for wound treatment because of their antimicrobial properties.^108^ Regarding AMR, alginate derivatives such as alginate oligosaccharides work in a two-fold manner.^109^ First, it affects the biofilm formed by the bacteria; it not only decomposes biofilms already made but also newly birthed ones by disrupting ongoing formation. Second, it acts in a synergistic manner with conventional antibiotics by increasing antibiotic efficacy, modulating biofilms, and increasing intracellular antibiotic concentrations.

#### 3.1.4 Cellulose derivatives

**Fig. 6:**
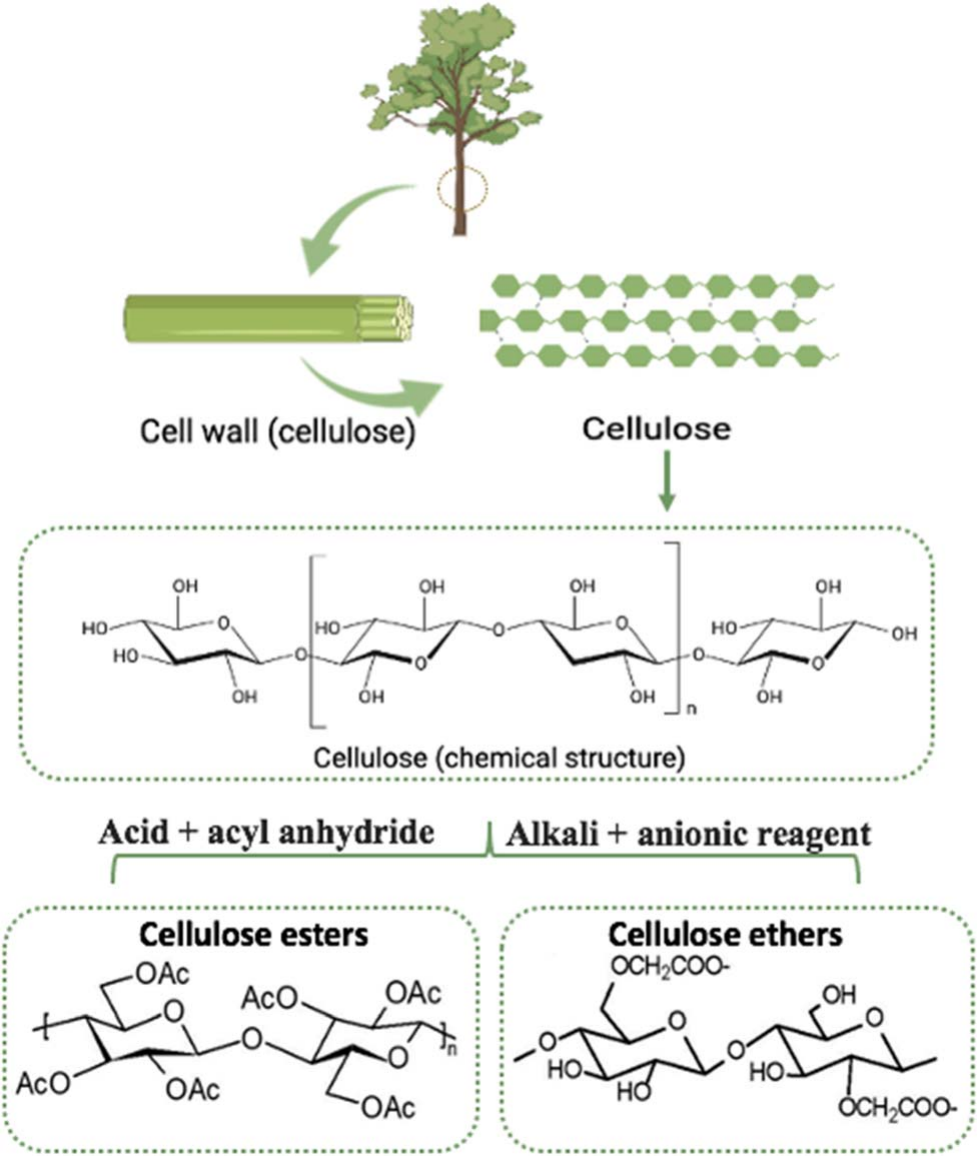
A schematic view of cellulose and its transformation into esters and ethers.

Cellulose is a polysaccharide composed of long links of glucose units joined by to 1–4 glycosidic bonds. It is the primary component of plant cell walls and the most abundant organic compound on earth.^11^ With chemical modification, cellulose derivatives have applications in various industries.^110^ Cellulose derivatives can be divided into two types based on their chemical modification: cellulose esters and cellulose ethers. The most common ester is cellulose acetate, which is commonly used in filters, drug delivery systems and films.^111^ In the second type, hydroxyl groups are replaced by ether groups; common examples are carboxymethyl cellulose (CMC) and methyl cellulose (Fig. 6). One derivative requires special mention, which is the modification of 1-methylimidazole. It showed remarkable antimicrobial effectiveness through mechanisms described previously. It also shows a promising future in AMR through advanced medical device coatings, novel drug delivery systems, improved wound care, and effective infection control measures.^112^

#### 3.1.5 Pectin

Pectin is a plant-based polysaccharide that is abundantly found in fruits and vegetables, particularly citrus peels and apples.^113^ Some of the more popular applications include use in jams and jellies as thickeners and gelling agents, but only recently have we begun to see a broader application in the biomedical and pharmaceutical sectors.^114^

One of the reasons why it is gaining attention is its biocompatibility, biodegradability, and low toxicity, which make it suitable for therapeutic formulations.^115^ Pectin has been used in wound healing patches, oral drug carriers, and even as a colon-targeting delivery agent because of its ability to remain stable in acidic gastric environments; however, under higher pH conditions of the colon, pectin degrades easily.^116^ Notably, pectin in its natural form does not have potent antimicrobial effects. To bring about this trait, it must be modified; only then does it exhibit a certain degree of antimicrobial activity. For instance, pectin, when combined with metal nanoparticles such as silver or copper, or when broken down into smaller fragments called pectic oligosaccharides (POS), levels up to gain a more pronounced antimicrobial activity.^117^ Moreover, these oligosaccharides have also been studied for their prebiotic activity, where they can be used selectively to supplement beneficial gut flora while counteracting pathogenic ones.^118^

Some researchers have experimented with methods such as pectin grafting and amidation.^119^ Going even further is the production of nanoscale drug delivery systems by combining pectin with antibiotics or antimicrobial peptides. These altered forms have been successful in breaking through bacterial biofilms and have demonstrated activity against some common multidrug-resistant strains such as *E. coli*, *S. aureus*, and *Pseudomonas aeruginosa*.^120^

#### 3.1.6 Natural proteins

While most biopolymers are carbohydrates in origin, natural proteins present a different category with their own pros and cons, particularly when it comes to biomedical applications. Among them, gelatin, silk fibroin, and collagen are the most widely used because of their structural similarity to human tissues, indicating a relatively low immunogenic response, which is crucial for patients who want to avoid side effects.^121,122^

Gelatin and collagen are derived from animal connective tissue, while silk is derived from *Bombyx mori* silkworms. The latter is comparatively more expensive but is unmatched in its mechanical strength and slow degradation rate; hence, it is suitable for long-term implants or sustained drug-delivery platforms.^123^ Gelatin is essentially a denatured form of collagen obtained through partial hydrolysis and is known for its excellent film-forming properties.^124^ It has been used in capsule making, soft gels, drug delivery coatings, and as a scaffold material in tissue engineering.^125^ On the other hand, collagen is a natural major structural protein in mammals; thus, its compatibility with skin and connective tissues is at its peak; this is why it is the ideal material for wound dressings as well as a matrix for cell growth.^126^ For example, the use of collagen matrices loaded with silver nanoparticles or antibiotics such as gentamicin has been reported in the literature; their efficacy lies in treating infected wounds, especially chronic wounds.^127,128^

Silk fibroin has also been used as a nanofiber mesh with embedded antimicrobial peptides; owing to its slow degradation profile, it allows for controlled release of the therapeutic agent, which is helpful for maintenance doses.^129^ It has also been used in cancer therapy.^130^ Gelatin films can be embedded in essential oils or metal oxides to form biodegradable antimicrobial packaging or wound care materials.^131^ One study even reported the use of a gelatin–silica hybrid loaded with dopamine to accelerate wound recovery.^132^ Some studies have explored crosslinking collagen with chitosan or grafting silk fibroin with quaternary ammonium groups to introduce positive charges that can interact with negatively charged bacterial cell walls.^133^

### 3.2 Synthetic biopolymers

**Fig. 7:**
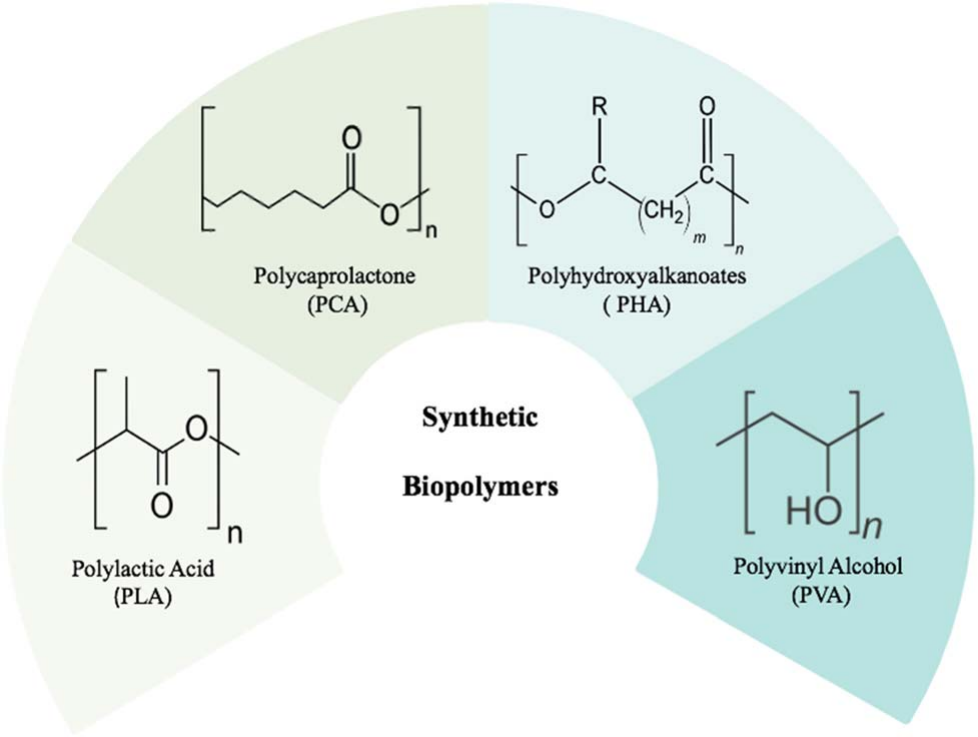
An illustration showing the structural diversity of synthetic biopolymers.

Synthetic biopolymers are polymers designed to mimic the properties of natural biopolymers. They offer numerous benefits, such as controlled structures, tunable functionalities, and improved mechanical stability. Some common examples include polylactic acid (PLA), polycaprolactone (PCL), polyhydroxyalkanoates (PHA), and polyvinyl alcohol (PVA); all of these are frequently utilized in biomedical applications are (Fig. 7).

#### 3.2.1 Polylactic acid (PLA)

Polylactic acid (PLA) is a synthetic aliphatic polyester that has garnered more attention over the years owing to its favorable properties, such as biodegradability, mechanical strength, and ease of derivation.^134^ Unlike some petroleum-based plastics that have long degradation timelines, PLA decomposes sooner under industrial composting conditions; the end products are water and carbon dioxide, which makes PLA an exceptionally eco-friendly option compared to other biopolymers.^135^ Although it resembles regular plastic and has been used in a variety of commercial packaging and 3D printing materials, its real value lies in the biomedical field.^136^

The biopolymer has undergone several toxicological assessments and has been deemed biologically compatible; this is why it has been explored for applications such as drug delivery nanoparticles, orthopedic screws, tissue scaffolds, and even sutures.^137^ Several new versions of composites have been devised to enhance the antimicrobial properties of PLA. Metal ions such as silver, zinc oxide, or copper are injected into the matrix to form nanocomposites, which have the ability to gradually release antimicrobial ions over time.^138^ Compared to other biopolymers, PLA has the greatest advantage of adaptability.^139^ Recent research has also explored PLA fibers incorporated with chitosan to create a dual-action material.^138^ This synergy between the two substances offers both structural strength and antibacterial effects, particularly against Gram-positive strains such as *Staphylococcus aureus*.^140^

#### 3.2.2 Polycaprolactone (PCL)

Polycaprolactone (PCL) is another synthetic polymer that carries with it its own favorable traits and has been steadily gaining ground in biomedical sciences.^141^ Because it breaks down over the course of months to years depending on its formulation, it has been used in long-term applications, such as drug implants, sutures, and tissue engineering scaffolds.^142^ Unlike naturally derived biopolymers, such as chitosan or gelatin, PCL does not possess any intrinsic antimicrobial capabilities from the start. This is only conferred upon after being modified and combined with other agents.^143^ One method involves electrospinning PCL into nanofibers. This expands its surface area and allows it to be coated easily with a sufficient dose of antibiotics, peptides, or even natural substances, such as essential oils.^144^

Studies have shown that PCL-based nanocarriers can be loaded with ciprofloxacin or vancomycin and retain prolonged release properties, which helps overcome resistance linked to erratic drug exposure.^145,146^ Moreover, PCL composites with materials such as graphene oxide or quaternized chitosan have shown better activity against biofilms, making them useful for developing anti-biofouling surfaces for implants.^147^ One of the drawbacks of PCL is that it has a relatively low melting point and slower degradation than other biopolymers, which can be a limitation of fast-acting antimicrobial systems.^148^

#### 3.2.3 Polyhydroxyalkanoates (PHA)

Polyhydroxyalkanoates (PHA) are a family of biopolyesters that serve as intracellular energy storage granules in bacteria.^149^ What makes them unique is, their monomer composition can be altered depending on the carbon source and microbial strain, giving rise to different forms, such as polyhydroxybutyrate (PHB), polyhydroxyvalerate (PHV), and their copolymers. PHAs are fully biodegradable and biocompatible, making them promising candidates for drug delivery and tissue regeneration.^150^ They have shown strong potential as platforms for antimicrobial delivery systems.^151^ Indeed, studies have reported that PHB nanoparticles loaded with ciprofloxacin or gentamicin were effective in reducing biofilm thickness and viability of *Pseudomonas aeruginosa* and *Staphylococcus aureus*.^152^

In addition, PHAs have been combined with chitosan and tungsten to produce composite dressings which carries a two-fold benefit: they not only deliver antimicrobial action but also support cell adhesion and wound healing.^153^ Because of their biological origin, there is also ongoing work into genetically modifying bacteria to produce PHAs that are pre-loaded with antimicrobials. This pre-built cache of antimicrobial-laden PHA from a virtually endless supply of bacteria may seem far-fetched, and despite promising findings, the whole process is still in the experimental stages.^154^

#### 3.2.4 Polyvinyl alcohol (PVA)

Polyvinyl alcohol (PVA) is a synthetic water-soluble polymer that has been used for many years owing to its excellent film-forming, emulsifying, and adhesive properties.^155^ PVA is interesting in the context of biomedical applications because of its biocompatibility and non-toxic nature, which makes it an ideal material for use in eye drops, wound dressings, and controlled drug release formulations.^156^ Indeed, it has been used as a base for hydrogels and nanofibers that can be embedded in antibiotics, metal nanoparticles, or natural antimicrobials such as curcumin and essential oils.^157^ Furthermore, because it dissolves in water depending on its cross-linking degree, it provides a flexible release profile for therapeutic agents. This quality makes it suitable for localized treatment strategies such as skin patches or wound closure.^158^

One of the notable strengths of PVA is its ability to form stable electrospun nanofibers.^159^ These fibers have a large surface-area-to-volume ratio, which improves contact with microbes and can increase the effectiveness of any incorporated antimicrobial substance. PVA nanofibers doped with silver nanoparticles or zinc oxide have been shown to be effective against common pathogens, such as *S. aureus*, *E. coli*, and even fungal strains, such as *Candida albicans*.^160^ Researchers have also experimented with blending PVA with other biopolymers, such as chitosan or gelatin, to create synergistic effects; this is an instance where the structural integrity of PVA is combined with the bioactivity of the other polymer.^161^ A complete summary of all biopolymers is presented in Table 2.

**Table 2:** Grand summary of biopolymers, modifications and their applications

Biopolymer	Origin	Basic structure	Biodegradability	Innate antimicrobial activity	Common modifications	Applications	Ref.
Chitosan	Natural	Linear polysaccharide of glucosamine units	High	Yes	Sulfation, carboxymethylation, nanoparticle embedding	Wound dressings, drug delivery, food packaging	162
Alginate	Natural	Anionic copolymer of mannuronic and guluronic acids	High	No	Crosslinking, blending nanoparticles	Hydrogels, dressings, water purification	163
Cellulose	Natural	Glucose-based linear polysaccharide	High	No	Carboxymethylation, amination	Films, coatings, filters, drug carriers	164
Pectin	Natural	Galacturonic acid backbone with methyl esters	High	Weak	Grafting, nanoparticle conjugation	Colon-targeted delivery, wound healing, prebiotic coatings	165
Gelatin	Natural protein	Denatured collagen; triple helix regions	Moderate	No	Crosslinking, hybrid nanofibers	Drug delivery films, implants, antimicrobial foams	166
Silk fibroin	Natural protein	Fibrous protein; β-sheet domains	Moderate	No	Blending with antimicrobial peptides	Long-term implants, sustained drug delivery	167
Polylactic acid	Synthetic	Aliphatic polyester from lactic acid	High	No	Nanocomposites, surface coating	Sutures, packaging, nanocarriers	168
Polycaprolactone	Synthetic	Semi-crystalline polyester	Slow	No	Blending nanoparticles, electrospinning	Nanofibers, membranes, long-term drug delivery	169
Polyvinyl alcohol	Synthetic	Water-soluble synthetic polymer	Partial	No	Embedding nanoparticles, blending with chitosan	Hydrogels, biosensors, wound healing films	156
Polyhydroxyalkanoates	Natural origin	Polyesters synthesized by bacteria	High	No	Functional nanoparticle loading	Biodegradable implants, antimicrobial membranes	170

## 4. Chemical modifications to enhance antimicrobial performance

Chemical modifications such as sulfation, carboxymethylation, phosphorylation, and amination of biopolymers enhance antimicrobial activity through several mechanisms including increasing charge density or enabling stronger interactions with microbial membranes; this in turn causes disruption of pathogens.^13^ On the other hand, to minimize cytotoxicity, besides the fact biocompatible functional groups are used (which are inherently safer) these modifications are meticulously added at a set limit weighing both pros and cons. Moreover, safer crosslinkers and optimal modification levels are chosen to retain host cell safety without compromising on the antimicrobial potency.

### 4.1 To improve solubility and microbial interaction

#### 4.1.1 Sulfation

Sulfation is a chemical modification process where sulfate groups (–SO_3_H) are introduced into the backbone of biopolymers, typically onto hydroxyl-containing substrates such as cellulose, chitosan, or alginate.^101^ This process not only increases water solubility by enhancing the hydrophilicity of the polymer but also significantly changes its biological activity.^171^ In particular, sulfated polysaccharides have shown improved antimicrobial, antiviral, and anticoagulant effects owing to their ability to mimic naturally occurring sulfated glycosaminoglycans, which can be found on cell surfaces.^172^

Regarding the antimicrobial aspect, the introduction of sulfate groups leads to a higher negative surface charge, which can interfere with the adhesion and communication of bacterial cells, which is useful for disrupting the early stages of biofilm formation.^173^ Some sulfated biopolymers have been reported to bind directly to bacterial adhesins or viral glycoproteins, thereby blocking their attachment to host tissues.^174^ Sulfated chitosan has shown boosted activity against both Gram-negative and Gram-positive bacteria when compared to its native form, while sulfated alginate has been reported to inhibit enveloped viruses such as HSV and influenza.^175,176^ The defensive coating conferred by them make them essentially ‘flak jackets’ against pathogens, as such, they are suitable for use on medical devices, wound dressings, or even filtration membranes in defense scenarios. Although sulfation can sometimes reduce mechanical stability or biocompatibility, depending on the degree of substitution, ongoing optimization studies are aimed at balancing these trade-offs.

#### 4.1.2 Carboxymethylation

Carboxymethylation involves the attachment of carboxymethyl (depicted as –CH_2_–COOH) groups into the polymer structure, which is typically accomplished by combining a biopolymer with monochloroacetic acid under alkaline conditions.^177^ This modification is most commonly applied to biopolymers such as cellulose and chitosan; the resulting end products being carboxymethyl cellulose (CMC) and carboxymethyl chitosan (CMCS) respectively.^178,179^

CMCS has shown increased activity against *S. aureus* and *E. coli* compared to native chitosan, particularly in moist wound environments; the water content of the wound makes CMCS a tactical choice in this regard.^180^ Furthermore, CMC has been used as a scaffold or hydrogel base to load AgNPs or antibiotics to form multifunctional composites with both mechanical strength and broad-spectrum antimicrobial action against UTI agents.^181^ In some cases, carboxymethylation also improves the film-forming ability of the polymer, which is useful in packaging and wound dressing applications.^182,183^ For instance, CMCS-based films have been studied as bioactive coatings on sutures or implants to prevent infection at the surgical site. While carboxymethylation generally improves functional properties, the process of over-substitution can lead to excessive swelling; therefore, the degree of substitution must be carefully considered and tailored on the intended use.

#### 4.1.3 Phosphorylation

Phosphorylation is a chemical modification in which phosphate groups (–PO_4_^3−^) are shifted onto the polymer backbone, usually hitting the hydroxyl groups through reactions involving phosphoric acid or phosphorus oxychloride.^184,185^ In antimicrobial applications, phosphorylated biopolymers have shown enhanced performance against both Gram-positive and Gram-negative bacteria.^186^ While the mechanism of action isn't clearly defined, one proposed idea is that it is possible that the increased negative charge allows for stronger interactions with cationic antimicrobial agents. Another proposed mechanism is the undoing of microbial cell walls through ionic imbalances. In hybrids, phosphorylated chitosan has been shown to be a coating material for wound dressings and implant surfaces.^187^ As with other modifications, the degree of substitution must be controlled to maintain mechanical integrity and biocompatibility, especially when used in systems requiring long-term implantation or contact with sensitive tissues.

#### 4.1.4 Amination

Amination refers to the chemical modification of a polymer by introducing primary or secondary amine groups (–NH_2_ or –NHR) onto its framework. This modification is often performed *via* reductive amination or amidation reactions. It is highly effective in enhancing the functional properties of polymers such as cellulose, starch, and chitosan.^188,189^ Regarding antimicrobial properties, amine-functionalized polymers should be highlighted as they excel in the ability to pierce through bacterial cell walls; they can do this by breaking down the components of the cell wall through electrostatic interactions.^190^ In some studies, aminated cellulose and starch derivatives have shown better antibacterial properties without requiring additional drug loading, especially against *E. coli* and *S. aureus*.^191^ In wound care materials and medical coatings, amination is used to create positively charged surfaces that resist bacterial colonization and biofilm formation.^192^ However, it is important to balance the degree of amination because an excessive positive charge can lead to increased cytotoxicity, making it unbearable for patients.

### 4.2 To enhance mechanical properties and stability

#### 4.2.1 Crosslinking

Crosslinking is a widely used technique that improves several areas: the mechanical integrity of a composite, thermal stability under extreme temperatures, and overall structural dependency of biopolymers, particularly those that are otherwise prone to breakdown in fluids.^193^ One of the most common crosslinking agents used for biopolymers is glutaraldehyde, which reacts readily with amino or hydroxyl groups found in natural polymers, such as chitosan, gelatin, and collagen.^133^ A crosslinked biopolymer tends to swell less and break down more slowly, which means a more controlled and sustained release of incorporated antimicrobials, such as silver nanoparticles, antibiotics, or essential oils.^194,195^ For instance, in wound dressings, this can help maintain therapeutic levels of antimicrobial agents over a number of days without requiring frequent wound dressing changes.

Furthermore, crosslinking can influence surface roughness and porosity; both these two properties are crucial, as they both can impact bacterial adhesion and biofilm formation.^196^ Some studies have shown that highly crosslinked hydrogels can reduce microbial colonization by simply creating a more compact surface that limits nutrient diffusion and microbial attachment.^197^ Thus, the type and degree of crosslinking must be carefully managed because excessive crosslinking, especially with agents such as glutaraldehyde, can lead to cytotoxicity, which can influence adherence in the long run.^198^ To address this, alternative crosslinking agents, such as genipin, which is a naturally derived compound, carbodiimide, or even ionic crosslinkers, such as calcium chloride (in the case of alginate), are being explored for applications where gentler modification is required.^199,200^

#### 4.2.2 Copolymerization

Copolymerization involves chemical union of two or more monomers into a single polymer chain.^17^ Natural polymers, such as chitosan or gelatin, are often copolymerized with synthetic polymers, such as PVA, PEG, or PLA, giving the end-product more flexibility, durability, and water stability. These synthetic components contribute to mechanical reinforcement, while natural components provide cell compatibility.^201,202^

Copolymerization also allows the implantation of charged groups, which can influence the antimicrobial interactions. For instance, copolymers of chitosan with quaternary ammonium-containing monomers have shown increased bactericidal effects; the electrostatic charges generated from them can disrupt microbial membranes.^203^ In some systems, the copolymer backbone is used to hold down antimicrobial agents such as peptides, metal ions, or photosensitizers for light-activated disinfection.^204^ One of the key advantages of copolymerization is its ability to accurately set the degradation rate and drug release profile of the material. This is done by adjusting the ratio and type of monomers; the created system not only degrades slowly over time but also resists environmental triggers, such as pH, temperature, or enzymatic activity. This makes copolymer-based systems highly suitable for long-term implants, chronic wound care, or even battlefield-ready antimicrobial dressings that need to remain effective under harsh conditions. However, challenges remain in ensuring biocompatibility and regulatory approval, particularly when synthetic monomers are used. The safety profile of the degradation byproducts has been the subject of discussion, which makes copolymer synthesis something that needs to be thoroughly evaluated before being reproduced in other clinical applications.

#### 4.2.3 Hybridization with nanofillers

Hybridization with nanofillers is the fusion of nanoscale materials into a biopolymer matrix to create composite systems. This amalgamation exhibits enhanced mechanical, thermal, and antimicrobial properties. However, nanofillers, ranging from metallic nanoparticles to carbon-based materials and metal–organic frameworks, act as reinforcing agents that also introduce functional capabilities.^15^ The resulting biopolymer nanocomposites offer a multilayered approach to combat resistance by combining physical barrier properties, controlled drug delivery, and direct antimicrobial action.^205^ Nanofillers can modify the biodegradation pathways of biopolymers in multiple ways.^15^ Firstly, they can change the speed of acceleration for degradation but this is largely dependent on their type, surface chemistry, and interaction with the polymer matrix. Secondly, if we consider hydrophilic nanofillers like nano clays, they can enhance water absorption and cause faster enzymatic breakdown. Thirdly, some nanofillers create more amorphous zones, allowing the polymers to fall readily to enzymatic attacks. In contrast, hydrophobic nanofillers like graphene can reduce water or gas diffusion and delay degradation.

Metallic nanoparticles such as silver (AgNPs), zinc oxide (ZnO), copper oxide (CuO), and titanium dioxide (TiO_2_) are among the most commonly used nanofillers, and the number of studies that have studied these in combination with other polymers is substantial, and the antimicrobial effects are well documented.^206^ Carbon-based nanomaterials, such as graphene oxide and carbon nanotubes, have also been rigorously analyzed. This is because in the process of hybridization, they tend to exhibit a unique surface area and an ability to generate reactive oxygen species, the latter of which can physically disrupt microbial membranes.^16,207^ This dual effect, when added to polymer films or coatings, provides not only strength and durability, but also passive antimicrobial activity, similar to a shield. It should be noted that the physicochemical properties of nanofillers strongly influence the release kinetics of antimicrobial agents.^15^ More surface area means more binding sites; higher porosity means more easy diffusion; surface charges can influence interactions; overall morphology can speed up or slow the release depending upon the exact shape and surface modification.

Hybridization also offers several anatomical benefits. Nanofillers often act as crosslinking points, meaning they help reduce brittleness and improve overall flexibility. This makes the material more suitable for real-world applications, such as wound dressings and surgical meshes that need to be manipulated around wounds and limbs. In addition, nanocomposites can be engineered to respond to stimuli such as light, heat, or pH, giving us a smart system that responds to a particular antimicrobial release based on specific environmental triggers. Despite their promise, challenges remain on several fronts. Indeed, the use of metals brings up discourse on the environmental friendliness of design. In addition, issues regarding uniform dispersion and long-term biocompatibility should be considered. A complete summary of the chemical modification techniques used is presented in Table 3.

**Table 3:** Summary of chemical modifications

Modification type	Chemical group added	Purpose	Common biopolymers used	Ref.
Sulfation	–SO_3_H (sulfonic acid group)	Improve solubility, enhances interaction with microbial membranes	Chitosan, alginate, cellulose	171
Carboxymethylation	–CH_2_COOH (carboxymethyl group)	Increase water solubility, improve antimicrobial loading and film formation	Chitosan, cellulose, starch	177
Phosphorylation	–PO_4_^3−^ (phosphate group)	Enhance hydrophilicity while allowing for electrostatic interaction with pathogens	Chitosan, cellulose	187
Amination	–NH_2_ or –NHR (primary or secondary amine group)	Adds positive charges for membrane disruption	Cellulose, starch, chitosan	189
Crosslinking	–CHO (sometimes can be aldehyde bridges, sometimes ionic bridges)	Increase mechanical strength, prolong drug release, reduce degradation	Chitosan, gelatin, alginate	193
Copolymerization	Varies	Customize mechanical and degradation properties	Chitosan, PLA, PVA	201
Hybridization with nanofillers	Embedding of nano metals (no covalent linkage formed)	Add antimicrobial activity and enables smart response	Chitosan, PVA, alginate	205

## 5. Types of nanofillers used to enhance antimicrobial and antiviral properties

**Fig. 8:**
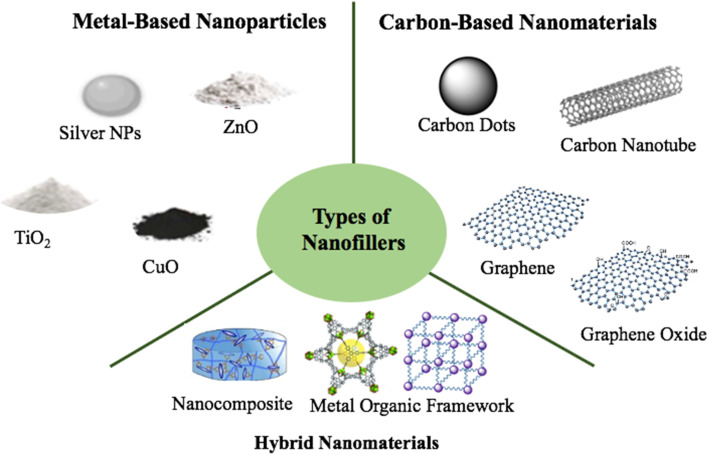
Overview of different types of nanofillers employed to enhance antimicrobial effectiveness.

Nanofillers are nanoscale materials incorporated into biopolymer systems to enhance their antimicrobial and antiviral properties. They are broadly classified into three main types based on composition and mechanism: metal-based nanoparticles, carbon-based nanomaterials, and hybrid nanomaterials. Each type offers distinct modes of action and advantages, making them valuable in developing advanced antimicrobial platforms (Fig. 8).

### 5.1 Metallic nanoparticles

Metallic nanoparticles are perhaps the most widely used nanofillers in antimicrobial biopolymer composites.^208^ In addition, metals exhibit high surface reactivity and disrupt microbial processes at multiple levels. In particular, silver nanoparticles (AgNPs) have been extensively studied and are often considered the gold standard for nanoantimicrobials.^209^ Their special uniqueness lies in their ability to inhibit bacterial growth at extremely low concentrations. Other commonly incorporated metallic nanoparticles include zinc oxide (ZnO), copper oxide (CuO), and titanium dioxide (TiO_2_), each of which has its own unique composite characteristics and mechanisms. Generally, the primary mode of action for most metallic nanoparticles involves several actions. These include either the generation of reactive oxygen species (ROS), disruption of microbial membranes, or interference with DNA/protein synthesis.^210^

Incorporating metallic nanoparticles into a biopolymer matrix has other advantages.^208,211^ These include stabilization while allowing for the sustained release of ions over time. Polymers such as chitosan, alginate, and PVA are commonly used as hosts to ensure that infused nanoparticles do not aggregate. For example, in wound dressings, AgNP-loaded biopolymer films have demonstrated not only strong antibacterial activity, but also promoted faster healing and reduced inflammation.^212^ Similarly, ZnO and CuO composites have been used in food packaging, surgical meshes, and antimicrobial coatings.^213,214^ Despite their effectiveness, metallic nanoparticles have drawbacks.^215^ Concerns have been raised regarding their cytotoxicity and environmental impacts. This is especially true when metals are used in high concentrations or in applications involving tissue exposure for an extended duration.

### 5.2 Carbon-based nanomaterials

Carbon-based nanomaterials are sometimes overshadowed by their metallic counterparts, but they deserve more attention than they usually receive. This group includes materials such as graphene oxide (GO), reduced graphene oxide (rGO), carbon nanotubes (CNTs), and carbon quantum dots, all of which have unique surface properties and nanoscale morphologies that render them suitable for antimicrobial applications.^216^ Graphene oxide, in particular, has been studied for its ability to cause direct damage to microbial membranes.^217^ It has sharp edges at the nanoscale that can physically slice through bacterial envelopes, and its high surface area allows it to adsorb proteins, lipids, or drugs. Carbon nanotubes share similar traits, with the added benefit of strong mechanical properties, making them suitable for reinforcing films or fibers.^218^ There is also some evidence that these materials can interfere with electron transfer processes in microbial respiration, although as of this writing, the exact mechanisms has not been elucidated clearly yet.^219^

One of the most notable characteristics of carbon-based nanomaterials is their tunability.^220^ The attachment of carboxyl, hydroxyl, or amine groups to the surface and dispersion in biopolymer matrices greatly improves, which in turn helps to increase their antimicrobial activity. Some composites have been designed to respond to pH or temperature changes by releasing embedded agents only when a set high or low pH or temperature is detected. This type of responsiveness adds another layer of control in applications such as smart wound dressings or anti-biofouling coatings.^221^ However, carbon nanomaterials are not entirely without problems.^222,223^ Their hydrophobic nature can lead to aggregation, especially in aqueous systems, and concerns have been raised about their long-term biocompatibility and potential cytotoxicity, particularly for CNTs.

### 5.3 Hybrid nanoparticles

Hybrid nanoparticles offer a different type of functionality compared to pure metallic or carbon-based fillers. The combination is a result of mixing biodegradable materials, such as PLA, PLGA, PCL, or naturally sourced polymers, such as chitosan and alginate, with metals. Their main appeal lies in enhanced controlled drug delivery. They can encapsulate antibiotics or antimicrobial peptides and shield them from early degradation.^152^ Some, such as chitosan nanoparticles, have inherent antimicrobial properties due to their positive surface charge, which disrupts bacterial membranes, as has been explained previously.^153^ When these nanoparticles are embedded into biopolymer films or gels, they provide dual benefits: mechanical support from the host polymer and bioactivity from nanofillers. This synergy is possible at the molecular level by the formation of strong bonds that can either be physical or covalent interactions.^204^ The advantage of this is that it improves dispersibility and stability of the compound. The combination is effective because nanofillers release metal ions or generate reactive oxygen species when coming into contact with microbes, while the biopolymer matrix provides sustained release of said nanofillers. This synergistic effect makes polymeric nanoparticles preferable, if feasible. Although they are generally more biocompatible than metal-based systems, polymeric nanoparticles face challenges in terms of scale-up and consistency. A quick summary of all the nanofillers covered is shown in Table 4, and their combinations with biopolymers are shown in Table 5.

**Table 4:** Comparative overview of nanofillers

Nanofiller type	Antimicrobial mechanism	Toxicity profile	Ref.
Silver nanoparticles	Ion release → membrane disruption, protein denaturation, ROS generation	Moderate to high	209
Zinc oxide	ROS generation, cell membrane destabilization	Low to moderate	213
Copper oxide	Ion-induced oxidative damage, enzyme interference	Moderate; potential cytotoxicity at high dose	214
Titanium dioxide	Photocatalytic ROS production under UV light	Low; generally considered biocompatible	224
Graphene oxide	Physical piercing of membrane, ROS production, adsorption of biomolecules	Low to moderate; depends on surface modification	16
Carbon nanotubes	Membrane insertion, oxidative stress, disruption of electron transport	Moderate to high; concerns over biopersistence	222
Metal–organic frameworks	Controlled ion release, surface functionalization, catalytic antimicrobial action	Variable	18

**Table 5:** Synergistic combinations of biopolymers and nanofillers

Biopolymer matrix	Nanofiller incorporated	Target microorganism/virus	Reported MIC/antiviral index	Ref.
Chitosan	Silver nanoparticles	*Escherichia coli*, *Staphylococcus aureus*	MIC: 1.6–3.1 μg mL^−1^	138
Chitosan	Zinc oxide nanoparticles	*Pseudomonas aeruginosa*, *Candida albicans*	MIC: 0.417–4 mg mL^−1^	225
Polyvinyl alcohol	Copper oxide nanoparticles	*Klebsiella pneumoniae*, *Staphylococcus epidermidis*	MIC: 62 μg mL^−1^	226
Gelatin	Graphene oxide	*Staphylococcus aureus*, *Escherichia coli*	∼45% reduction in viral titer	227
Alginate	Titanium dioxide	Influenza A, HSV-1, SARS-CoV-2	∼87% reduction in viral titer	228
PCL/PLA blend	Silver + ZnO hybrid	MRSA, *Escherichia coli*, *Acinetobacter* spp.	MIC: 5–15 μg mL^−1^	229
Chitosan/PEG	Metal–organic frameworks	*Staphylococcus aureus*, *Escherichia coli*	22.5 mm *vs.* 14 mm (*E. coli vs. S. aureus*)	230

### 5.4 Safety concerns & toxicity

There are a number of concerns regarding the safety of nanocomposites.^208,215^ The safety concerns include but are not limited to the following: bioaccumulation, cytotoxicity, and body interactions. The former refers to the accumulation of nanoparticles in tissues. The buildup can potentially be toxic unless it is metabolized or excreted. Cytotoxicity is mostly due to oxidative stress caused by ROS, and nanofillers are well known to generate these. The lattermost safety concern refers to how nanofillers can interact with cells, tissues, and even the immune system. This can trigger immune responses hypersensitivities or even inflammation *via* a number of mechanisms. However, there is also lack of insufficient mechanistic insight into how nanoparticle properties drive immune activation. Minor topics that aren't covered well include the roles of protein corona formation, complement activation, and antibody generation, especially with commonly used coatings like PEG.

Nanocomposite antimicrobial agents may have off-target effects on human cells as well. To enlist, these include cytotoxicity, oxidative stress, membrane damage, and disruption of DNA.^204^ Additionally, these nanocomposites can disrupt the natural flora causing dysbiosis. Besides this, some nanocomposites, such as those containing copper or zinc oxides, have shown build up in organs like the liver and kidneys, leading to damage and ultimately chronic disease. While some formulations, such as biogenic silver nanoparticles, have shown low toxicity, others have caused acute or chronic toxicity, particularly at higher concentrations. Ultimately, the toxic effects can vary widely based on the nanomaterial composition, size, dose, and duration of exposure.

Then we must also consider the effects to the environment.^55^ The use of nanomaterials poses several environmental risks, especially in resource-limited settings. As previously mentioned, these include bioaccumulation and persistence in soil and water, cytotoxicity to organisms, and contamination of local food webs. It doesn't help that currently, regulatory frameworks for evaluating and approving nanocomposite antimicrobial materials are not yet fully effective. This is because legal regulations and guidelines, such as those of the FDA and EMA, are mainly designed for conventional drugs or medical devices. This is why there is uncertainty about toxicity assessment, risk analysis, product classification, and environmental impact.

### 5.5 Challenges

There are a number of barriers that prevent the maximum utilization of nanoparticles.^273^ These include production at a grander scale as well as maintaining stability of the composite during distribution.

Firstly, when considering production at an industrial scale, there is no fine control over particle size and uniformity. This can affect both efficacy and safety. Secondly, maintaining consistency is also a challenge, as the complex multi-component systems are sensitive to small variations in process conditions. Thirdly, at a larger scale it becomes hard to purify the composites and remove solvents. Fourthly, storage issues can be a problem for those composites that are kept longer than the shelf-life; the loss of function is hard to gauge. Lastly, given the global economic hurdles, certain materials may have a high cost; even the equipment needed to work with these can be expensive. To maintain production quantity without compromising on quality, a number of steps can be taken. Taking nanoparticles for example, measures that can be taken include using advanced dispersion techniques to ensure uniform nanoparticle distribution, employing surface modification of fillers, adopting hybrid composite designs with multiple fillers for synergistic effects, and integrating process automation and AI/machine learning.

Strategies to enhance the stability and shelf life of nanocomposite antimicrobial products include a number of processes.^273^ The nanoparticles can have an antimicrobial agent encapsulated, such as in nanoclays, to control the release rate and prevent rapid degradation. The nanoparticles can be combined with biopolymers which improve its overall stability and moisture resistance. Moreover, surface treatments can provide useful functional groups that prolong the shelf-life.

Successful translation of nanocomposite antimicrobial agents into clinical and defense applications relies on extensive interdisciplinary collaboration among a number of individuals.^258^ These are as follows: materials scientists, chemists, biologists, toxicologists, clinicians, pharmacologists, regulatory experts, and military personnel themselves. A weakness in this linkage or a lack of contribution from any discipline can lead to compromises in quality and function. Moreover, materials scientists can work towards improvements to nanocomposites with the help of computational modeling. This aids understanding and prediction in nanocomposite systems through the use of simulations. The simulations can show biological interactions which can help in optimizing the end product. Furthermore, there are a number of innovative strategies that can be employed by biotechnologists. One example is the green synthesis methods, where biosynthesis of silver nanoparticles is done using plant extracts. These are more ecofriendly and do not eschew any positive trait. Scientists are also working on nanorobotics, which is a promising step that can revolutionize antimicrobial platforms. Basically, it allows enabling active, targeted delivery of antimicrobial agents directly to infection sites, especially in hard-to-reach regions. The nanorobots can autonomously navigate and penetrate biofilms to release antimicrobial payloads precisely where needed. This is a precision that is unprecedented but still being workshopped.

### 5.6 Limitations of nanocomposites

The durability of nanocomposite antimicrobial systems is variable. While many nanocomposite coatings exhibit strong mechanical stability and resistance to routine wear, abrasion, and wet cleaning, too aggressive abrasion or prolonged exposure to strong disinfectants can wear down the coating. This in turn can cause an overall decrease in antimicrobial efficacy.^236^

When it comes to environmental dynamics, if we consider humidity, this can enhance water adsorption within nanocomposite films, which in turn can influence electrical conductivity. While this may suggest higher humidity leads to increased reactivity, it should be noted that excessive moisture can cause structural rearrangements; this alone may decrease antimicrobial efficiency over time. Regarding temperature, this affects both the stability and bioactivity of nanocomposites. While moderate increases in temperature can speed up antimicrobial action, extremes of temperatures can reduce overall activity. This is understandable as higher kinetic energies can cause degradation of the nanocomposite. There are workarounds to improve thermal stability such as the incorporation of stabilizing agents such as ZnO.

Nanocomposite antimicrobial platforms often have higher initial costs, mostly due to complexities in the synthesis process, the need for specialized materials as well as sufficient quality control.^273^ But the long-term gains outweigh these initial costs. Such as nanocomposites having greater efficacy and lower resistance compared to conventional antibiotics. There is also the benefit of longer-lasting antimicrobial activity at lower doses. The spectrum of activity is comparatively wider, and most nanocomposite coatings, films, or wound dressings often remain effective longer, requiring fewer replacements.

As of this writing, there have been very few recorded cases of microbial resistances developing against nanocomposite-based antimicrobial platforms.^6^ This is largely due to the robust mechanism of actions provided by nanomaterials. Even so, some adaptive responses have indeed been observed, such as certain microbes undergoing changes in membrane protein expression to counter nanoparticles, though these aren't permanent or even stable.

## 6. Applications of modified biopolymer nanocomposites

**Fig. 9:**
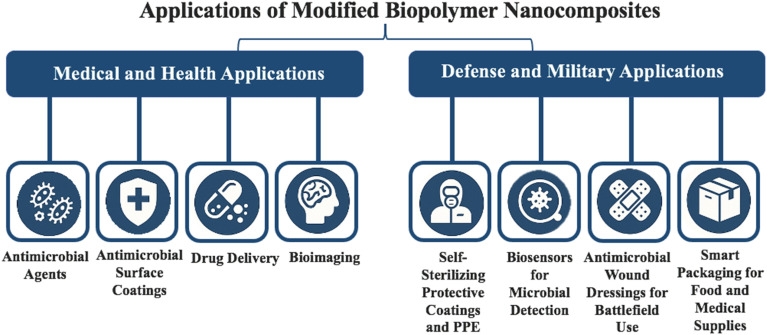
Diagram representing various applications of modified biopolymer nanocomposites.

Modified biopolymer nanocomposites are classified into two primary application domains: medical and health, and defense and military. This classification highlights their multifunctionality in areas such as wound care, bioimaging, smart packaging for food and medical supplies, and protective systems for high-risk environments (Fig. 9).

### 6.1 Medical and health applications

#### 6.1.1 Antimicrobial agents

Among the various uses of modified biopolymer nanocomposites, one of the most straightforward is their role as antimicrobial agents. Here, the material either acts directly against pathogens on its own or acts as a carrier or scaffold for other antimicrobial substances.^231^ Some biopolymers, such as chitosan or those modified with sulfate or amine groups, have their own ability to interfere with bacteria.^201^ Others, like PLA or PCL, do not do much on their own but serve as a base structure; when mixed with silver or copper nanoparticles or essential oils, the final product shows improved antibacterial action, often enough to handle resistant strains.^229^

One benefit of using biopolymers is their ability to slow down or sustain the release of antimicrobial components.^232^ Instead of getting a strong burst of activity at the beginning and then nothing, here the composite can keep working for hours or days, depending on how it is built. This is very useful for surgical meshes, wound dressings, or even dental scaffolds where long-term infection control is needed. Interestingly, progress has also been made in the field of environmentally sensitive release.^233^ This means that the antimicrobial payload is released only when certain conditions are met, such as low pH, temperature, or the presence of a specific enzyme. These “smart materials” are still being worked on, but they offer some promise in reducing side effects and making the response more focused. Lastly, some studies have shown that not only do these biopolymer-based systems reduce the bacterial count but they also help reduce local inflammation and accelerate tissue repair, especially in chronic wounds.^234,235^

#### 6.1.2 Antimicrobial surface coatings

Another area where modified biopolymer systems show considerable promise is in surface coatings, whether it is for medical devices, implants, door handles in hospitals, or even things as simple as surgical tools.^236^ The idea is to apply a thin layer of material onto a surface so that it either prevents bacterial adhesion from the start or kills off anything that attempts to settle on it. In most cases, the coating is made from biopolymers such as chitosan, gelatin, or alginate, but these are often combined with other additives, such as silver nanoparticles, quaternary ammonium compounds, or even essential oils.^237–239^ Some materials, particularly those with a positive charge, actively repel or damage the bacterial cell wall by interfering with the membrane integrity. Others, such as those based on PVA or PLA, do not do much on their own but can be loaded with slow-releasing antimicrobial compounds to create a passive but continuous barrier.^240^ Interestingly, some researchers have explored the concept of light-activated or thermoresponsive coatings.^241,242^ To simplify, these coatings remain dormant until they are triggered by specific environmental cues, such as high or low temperature. For example, some composites can release antimicrobial peptides when exposed to body heat or UV light. This type of control could be helpful for reducing the number of active agents required.

#### 6.1.3 Drug delivery

The use of biopolymers in drug delivery is not a new concept, but in antimicrobial therapy, especially in the context of resistance, these materials play a more critical role.^243^ Instead of acting as passive carriers, modified biopolymers now function as active participants in controlling how and when the drug is released, how much of it is delivered, and, more importantly, whether it even reaches the intended site at therapeutic levels. One of the most common examples is the encapsulation of antibiotics, such as ciprofloxacin or vancomycin, inside nanoparticles made from PLA, PCL, or PHB.^145^

Chitosan, with its natural mucoadhesive property, has been a favorite for drug delivery in mucosal tissues, such as the gut, nasal cavity, or even oral infections.^98^ When used in combination with other polymers such as alginate or gelatin, the drug release profile becomes more customizable, which helps in treating both systemic and localized infections.^244,245^ There is also ongoing interest in stimuli-responsive systems, in which drug release is tied to environmental conditions such as pH or enzymatic activity. For example, in infected wounds, where the pH is slightly acidic, some drug-loaded hydrogels begin to swell and release their contents only when these changes are detected.^246,247^ These platforms reduce the need for systemic antibiotics and allow site-specific delivery, lowering both the dose and toxicity.

#### 6.1.4 Bioimaging

While most attention on biopolymers has gone to their antimicrobial roles, there is a smaller but growing interest in their use in bioimaging, especially in cases where infection tracking or pathogen detection is needed. This concept works by attaching contrast agents, fluorescent dyes, or metal-based nanomaterials to a biopolymer structure, usually nanoparticles or hydrogels, and then guiding them to the infection site.^248,249^ Materials such as PLA, PCL or PEGylated chitosan are commonly used here, not because they kill bacteria directly, but because they provide a safe and biodegradable structure to carry these imaging agents.^250,251^

Some systems are dual-functional, meaning they act as both therapeutic and diagnostic tools a concept often referred to as theranostics.^252^ For example, chitosan nanoparticles might carry both an antibiotic and a fluorescent tag.^253^ It is taken up at the infection site, delivers the drug, and simultaneously gives off a signal that can be picked up by imaging tools such as MRI, CT, or near-infrared spectroscopy. This can help clinicians monitor how well a treatment is working or how far an infection has spread. There is also interest in using metal-based fillers, such as iron oxide or gold nanoparticles, within the biopolymer matrix.^254^ These not only provide a clearer imaging contrast, but in some cases, can be used to generate heat through magnetic or photothermal activation, which means that the system can also be used to kill bacteria through heat, in addition to visual tracking.

### 6.2 Defense and military applications

#### 6.2.1 Self-sterilizing protective coatings and PPE

Prevention is a key counterstrategy against infections in the military and defense settings. This is especially true in environments where access to proper sterilization or fully equipped medical facilities is limited or worsened during wartime.^255^ One effective approach that has been the subject of much study is the use of self-sterilizing biopolymer coatings, especially for protective gears such as gloves, uniforms, masks, and face shields.^256^ These coatings are typically made using biopolymers such as chitosan or alginate; the reinforcing agents are typically metallic nanoparticles such as silver, copper, or zinc oxide.^228,237^ The idea is to create a surface that neutralizes any microbe on first contact without the aid of a disinfectant. This is particularly useful in crowded field conditions, mobile units, or combat zones where the risk of pathogen transmission is exponentially high.^257,258^

Some systems rely on slow ion release to maintain antimicrobial action over time, while others are designed to be activated by light, temperature, or even humidity, all of which are in response to the varying environments and settings where unit or military personnel can be deployed.^259^ For example, a silver–chitosan hybrid coating might remain inert in dry air but become active once exposed to sweat or moisture, releasing ions and blocking microbial colonization; a combatant shifting into a tropical zone like the South American Jungles would be equipped with such a coating. Even if proper washing or disposal is not possible, the coated surface still works in the background to reduce the microbial load.^260^ This is particularly important when dealing with resistant strains or biohazard threats where conventional cleaning methods do not completely eradicate microbes. Overall, nanocomposite-enhanced biopolymers significantly improve the mechanical properties for protective military gear. It confers higher strength-to-weight ratios, improved toughness, increased impact resistance, enhanced flexibility and thermal stability, all of which are crucial to withstand extreme environments and situations.^197^

#### 6.2.2 Biosensors for microbial detection

Biosensors made from biopolymers have recently emerged as a niche for defence applications. Unlike laboratory diagnostics, which require time, equipment, and trained personnel, biosensors are meant to work on-site with little to no expertise and handling. The advantage of military personnel is that they can obtain real-time information about pathogens from the environment, equipment, or even the human body swiftly, thereby responding appropriately to avoid such pathogens.^261^ These components can be divided into three main parts.^262,263^ The first is biological recognition elements, such as antibodies, enzymes, or aptamers. Second, the biopolymer-based matrix acts as a scaffold or platform to host the element. Third, the transducer, which is linked to the former two, converts the microbial detection event into a readable electrical, optical, or colorimetric signal.

Selected materials such as chitosan, cellulose, or PVA hydrogels are often used because they possess a number of perks.^264–266^ On one hand, they are easy to process and can be manufactured cheaply and quickly. Moreover, they have functional groups that allow for the binding of the sensing elements. For example, chitosan-encapsulating flavonoids can detect *E. coli* in biofilms within minutes.^267^ Some biosensor platforms are tailored for wearable devices, such as patches or inserts in uniforms. They can monitor body fluids, such as sweat, for early signs of infection.^266^

#### 6.2.3 Antimicrobial wound dressings for battlefield use

Infection control is a top concern in battlefield injuries. Wounds sustained in combat settings are often exposed to soil, debris, and unclean tools, not to mention the fact that evacuation or treatment might be delayed for hours.^255^ In such cases, conventional wound dressings have several disadvantages.^268^ They dry out easily, often become a source of contamination, or do not provide any or sufficient antimicrobial activity on their own. Biopolymer-based wound dressings can serve to rectify this.^269^ These systems offer biocompatibility, moisture retention, and controlled antimicrobial agent release; all of which are key for successful wound healing. Additionally, many biopolymer matrices, such as chitosan and alginate, are non-toxic, biodegradable, and support tissue repair.^239^ Besides that, nanofillers provide sustained antimicrobial action even complex wound microenvironments. Some composites are more innovative as they respond to wound pH or enzymes to deliver drugs more effectively.^246^ On the other hand, it is still unknown how complex wound conditions would affect outcomes, so more studies are needed in this regard.

**Fig. 10:**
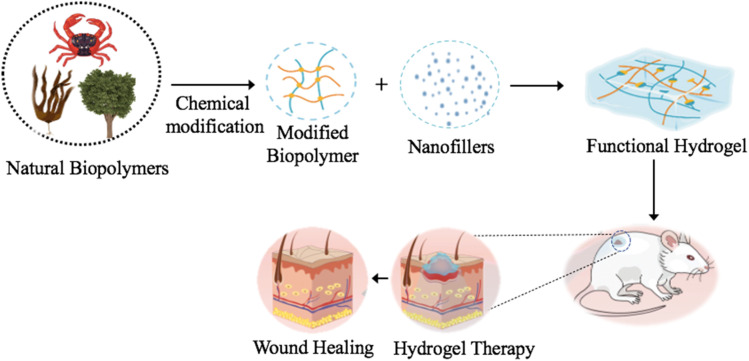
Biopolymer-based hydrogel, chemically modified and infused with nanoparticles for the purpose of wound dressing.

Materials such as chitosan, alginate, collagen, and gelatin, especially when modified or combined with nanoparticles, have shown significant promise in preventing infections while boosting the healing process. Chitosan, in particular, is known for its hemostatic properties, which make it useful for stopping bleeding quickly. When combined with silver or zinc oxide nanoparticles, the dressing can also be actively suppressed if microbes are not killed (Fig. 10).

Currently, these dressings are in the form of hydrogels, films, sponges, or electrospun fibers, and they can be made to release antimicrobial agents gradually over several days.^270^ Some advanced versions also include pH-responsive features, where the dressing changes color to indicate the presence of infection or begins releasing the drug only when the wound environment becomes acidic. These dressings offer the dual action of antimicrobial action and prohealing.^271^ In military use, these smart dressings excel conventional bandages, can easily be pre-packed in field medical kits, require no special training to apply, and serve as the first line of defense before a wounded soldier can reach a hospital.

Regarding rheological behavior (flow and deformation), the higher the better. Wound dressings such as hydrogels, films or electrospun fibers should be able to maintain integrity under stress while conforming to irregular wound surfaces; hence, they should have the ideal rheology.^268^ To illustrate, wound dressings benefit from having controlled viscosity and gel-like behavior to help retain moisture. This allows them to not only adhere gently to the wound bed but also promote healing. In filters, rheological properties affect the formation of uniform, porous structures required for efficient filtration and antimicrobial action. Overall, these dressings offer a low-tech, high-impact solution that aligns perfectly with frontline needs, especially when dealing with multidrug-resistant organisms, which are becoming increasingly common in combat zones.

#### 6.2.4 Smart packaging for food and medical supplies

In defense and military operations, clean and uncontaminated food and medical supplies are placed at the forefront of any operation because spoilage, microbial contamination, or unnoticed degradation can significantly impact operational safety, potentially hampering the overall effectiveness of the army or unit.^272^ To address this, smart packaging made from biopolymers is being explored as a means of extending the shelf life, improving safety, and providing real-time feedback on the status of stored items.^273^ The core idea is to create packaging films or coatings that are not just containers that prevent breaches by microbes but also active systems.^274^

Some forms of smart packaging go a step further by being responsive. To illustrate, pH-sensitive dyes or indicators that change color when the contents begin to spoil or degrade are incorporated.^275^ Having a visible built-in signal that indicates whether an item is still usable can reduce waste, prevent infections, and avoid logistical errors. In harsh or remote environments, this kind of early warning system expediates decision-making on where and how to obtain nutrition. A summary of tools utilized in defense and military applications of biopolymer nanocomposites is presented in Table 6.

**Table 6:** Applications of biopolymer nanocomposites in defense and military setting

Application	Materials used	Target microbial agents	Defense relevance	Ref.
Personal protective equipment	Chitosan + AgNPs/ZnO/CuO coatings	*E. coli*, *S. aureus*, influenza virus	Reduces surface contamination in masks, gloves, uniforms; prevents fomite transmission	276
Wound dressings	Alginate/gelatin hydrogels with silver or TiO_2_ nanoparticles	*Pseudomonas aeruginosa*, MRSA, *Klebsiella* spp.	Controls infection in battlefield wounds; promotes faster healing	212
Air filtration systems	Electrospun PLA/PVA nanofibers with ZnO, CuO, or GO	Bioaerosols	Protects confined spaces (tents, vehicles); enhances respiratory biosafety	144
Smart packaging	Cellulose/PVA films with essential oils, AgNPs, pH indicators	Spoilage bacteria, *Listeria*, fungal contaminants	Extends shelf-life; detects spoilage or contamination in field rations and supplies	274
Wearable biosensors	Chitosan/PVA matrices with antibody or aptamer-functionalized sensors	*S. aureus*, *E. coli*, viral RNA/protein targets	Early infection detection in soldiers; monitors exposure in real-time	266
Decontamination coatings	Alginate + TiO_2_ or AgNP spray films	Broad-spectrum	Used for sanitizing medical equipment, gear, or contact points in temporary facilities	277

#### 6.2.5 Ethical concerns

Deploying nanomaterials in both military and civilian contexts involves several key ethical considerations that must be highlighted. Firstly, there is the dual-use risk; nanotechnologies can be repurposed for harmful applications, such as bioterrorism.^5^ The environmental^55^ impact has also been previously mentioned, as well as the risk to human health. Besides this, there is the issue with privacy and surveillance; in both settings, nanoscale sensors enable advanced surveillance, potentially infringing on individual rights and privacy. Because of the integration of nanotech into autonomous military systems, this does raise concerns about human control and accountability and scenarios depicting AI initiating or escalating wars cannot be overlooked.^258^

## 7. Challenges and limitations

While the use of modified biopolymers in antimicrobial applications offers unprecedented advantages, there are still a number of challenges that need to be addressed before widespread adoption in military and defense settings can be realized.^278^ The first and foremost issue is scalability. Many of these materials, while promising in lab-scale studies, are difficult to procure or manufacture in bulk without compromising quality or consistency. Certain steps require a minimum but regular supply of specific materials to satisfy the processes of surface functionalization, nanoparticle loading, or crosslinking. Even minute variations in the formulation process can translate to significant differences in the antimicrobial performance, drug release rates, or physical properties of the final product. Another concern is biosafety.^279^ Although most biopolymers are biodegradable and safe, they cannot always be used as additives. Metal-based nanofillers, for example, can show cytotoxic effects if ion release is not properly controlled, particularly silver, copper, or titanium dioxide.^280^ Prolonged exposure or accumulation in tissues can lead to side effects, especially when these materials are used in implants, wound dressings, or respiratory applications. To confound the situation further, there is still limited data on the long-term degradation behavior of these composite systems once they are introduced into the human body or even the environment.^281^ Regulatory hurdles cannot be ignored.^282^ Any material intended for therapeutic use must pass through a series of toxicological, clinical, and environmental safety assessments, which can be time consuming and expensive. This makes it harder for experimental materials, especially those still in the development phase, to move toward commercial or defensive use. Another limitation is related to the storage.^283^ Some biopolymer-based systems are highly sensitive to humidity, temperature, and ultraviolet (UV) exposure. If a material is kept in an environment that does not match its minimum requirements for stability, it can lead to a breakdown of the product or a reduction in the activity of embedded antimicrobials. In military deployments, where conditions are extreme and refrigeration is not always available, this is a real concern. Ensuring shelf stability without compromising activity is a challenge that requires further investigation. Finally, there is an issue of resistance. Although many of these systems offer non-specific mechanisms of killing, the possibility of microbial adaptation cannot be ruled out. Microorganisms can evolve constantly. Just as the misuse of antibiotics can lead to the survival of resistant species, the same can occur with misuse or overuse of these products. This study would have also benefited from making comparative analysis between more robust studies, but the lack of RCTs and comparative studies made conducting a deeper investigation difficult.

## 8. Conclusion and future directions

The fight against AMR has reached a point where conventional strategies are becoming obsolete. This is the worst-case scenario, and the evolution of pathogens seems unstoppable. Thus, while existing antibiotics are losing their advantages, it is pertinent to adopt new approaches. This is not just for those on the medical frontline to implement, but the issue needs to be addressed at the legislative level, as the military and defence aspects of a country form one of its strongest pillars. Preventing their growth, improving drug delivery, and reducing their chances of resistance formation are key targets. In this regard, biopolymer-based materials, especially those modified with antimicrobial agents or used as nanocomposites, should be the focus of concerted efforts in the field of microbiology. From wound dressings to surface coatings, and from filtration systems to smart biosensors, the applications of these materials are already wide-ranging but must pass through rigorous testing and trials to fine-tune them sufficiently for maximum effect. The versatility of these tools is only matched by the fact that they are often biodegradable, biocompatible, and can be tuned according to the need. Whether through a slow yet constant drug release, dynamic pH response, or membrane disruption, these systems are already proving their worth in early stage trials. In military contexts, where access to clean facilities, timely interventions, and sterilized environments cannot always be provided to soldiers or staff, these materials offer a novel yet portable solution that conventional systems fail to provide.

Future research should focus on optimizing these composites. A number of deficiencies need to be addressed, such as making these composites suitable for long-term use, improving their environmental safety profile, and ensuring consistent performance on a large scale on demand. Efforts should also be directed at making these systems affordable, although this may lie outside the scope of this review, since military and defense budgets are highly variable from country to country, depending on the geopolitical situation. Even so, there is a need to combine these materials with artificial intelligence and biosensing platforms to create real-time responsive systems that adjust their behavior based on microbial load, patient status, or environmental conditions. This novel integration could redefine the detection, treatment, and monitoring of infections, which should be explored in future prospective studies. Regulatory clarity is another frontier. Many of these systems lie in a grey zone between biomaterials, pharmaceuticals, and diagnostics, which complicates their approval. Streamlined guidelines specific to hybrid or nanocomposite materials will be necessary to ensure that these tools make it beyond the research phase, and medical professionals in concert with military professionals will need to conduct joint exercises in this regard. Policymakers and regulatory agencies play a crucial dual role in advancing innovation while ensuring the safety and efficacy of nanocomposite. Simply put, they do this by establishing adaptive regulatory frameworks. These frameworks set clear safety standards and require thorough risk assessments before market approval. Agencies such as the FDA, EPA, and their counterparts are involved in creating these frameworks. Policymakers also help foster innovation by funding research as well as developing flexible guidelines.

In the end, while biopolymers may not replace antibiotics, they represent a strong complementary strategy until more efficacious or sustainable solutions are developed. If developed thoughtfully and applied with proper foresight, they could become a central component of infection control, both in civilian healthcare settings and on the frontlines.

## Conflicts of interest

The authors declare that they have no conflicts of interest.

## Data Availability

No primary research results, software or code have been included and no new data were generated or analysed as part of this review.
